# Crystalline AuNP-Decorated Strontium Niobate Thin Films: Strain-Controlled AuNP Morphologies and Optical Properties for Plasmonic Applications

**DOI:** 10.1021/acsanm.3c00934

**Published:** 2023-06-20

**Authors:** Qiaomu Yao, Andrey V. Berenov, Ryan Bower, Bin Zou, Xiaofei Xiao, Neil M. Alford, Rupert F. M. Oulton, Peter K. Petrov

**Affiliations:** † Department of Materials, Imperial College London, London SW7 2AZ, United Kingdom; ‡ Department of Physics, Imperial College London, London SW7 2AZ, United Kingdom

**Keywords:** pulsed laser deposition, gold nanoparticles, perovskite, plasmonics, strain engineering, strontium niobate, gold decoration, structural control

## Abstract

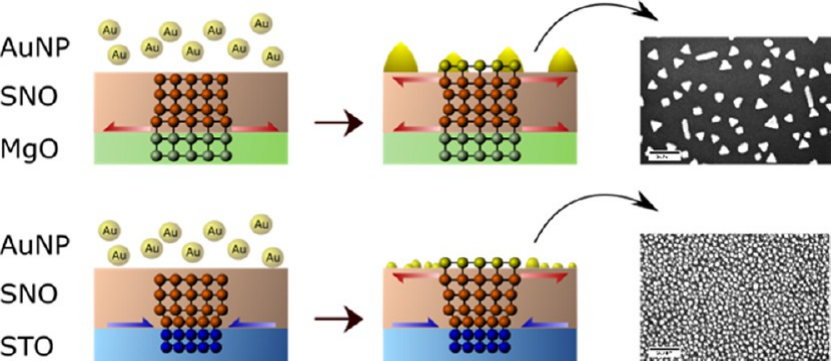

Gold nanoparticle (AuNP) decoration is a commonly used method to enhance the optical responses in many applications such as photocatalysis, biosensing, solar cells, etc. The morphology and structure of AuNPs are essential factors determining the functionality of the sample. However, tailoring the growth mechanism of AuNPs on an identical surface is not straightforward. In this study, AuNPs were deposited on the surface of a perovskite thin film, strontium niobate (SNO), using pulsed laser deposition (PLD). AuNPs exhibited a dramatic variation in their growth mechanisms, depending on whether they were deposited on SNO thin films grown on magnesium oxide (SNO/MgO) or strontium titanate (SNO/STO) substrates. On SNO/MgO, the Au aggregates form large NPs with an average size of up to 3500 nm^2^. These AuNPs are triangular with sharp edges and corners. The out-of-plane direction of growth is favored, and the surface coverage ratio by AuNPs is low. When deposited on SNO/STO, the average size of AuNPs is much smaller, i.e., ∼250 nm^2^. This reduction in the average size is accompanied by an increase in the number density of NPs. AuNPs on SNO/STO have a round shape and high coverage ratio. Such an impact from the substrate selection on the AuNP structure is significant when the sandwiched SNO film is below 80 nm thickness and is weakened for 200 nm of SNO films. X-ray diffraction (XRD) and scanning electron microscopy (SEM) were used to characterize all samples. Strain analysis was used to explain the growth mechanism of AuNPs. The average height of AuNPs was measured by using atomic force microscopy (AFM). Ellipsometry in the visible–near-infrared (vis–NIR) region was used to characterize the optical response of all samples. AuNP-decorated SNO/MgO and SNO/STO thin films exhibit different optical properties, with only gold-decorated SNO/MgO samples showing a size-dependent epsilon-near-zero behavior of nanoparticles. These results provide an additional route to control the structure of AuNPs. They can be used for various plasmonic applications like the design and development of strain-engineered gold-nanoparticle-decorated devices for surface-enhanced Raman spectroscopy (SERS) and photocatalysis.

## Introduction

Metallic nanoparticles decorated with thin films have been extensively studied and exhibit promising performance in a wide range of applications, including biosensing,^1,2^ electrical devices,^3^ photocatalysis,^4^ and solar cells.^5,6^ For perovskite thin films, the decoration can produce an enhanced plasmonic response, which indicates a promising future in nano-waveguides,^7^ energy harvesting,^8^ and chemical sensing^9^ applications. The plasmonic performance is strongly governed by the morphologies and chemistries of nanoparticles (NPs). For example, depending on the density and size of the embedded gold NPs (AuNPs), LiNbO_3_ thin films exhibited tailorable plasmonic responses, which have been demonstrated in silicon-based waveguide applications.^7^ Furthermore, the size and density of AuNPs dramatically influence the performance of the devices by changing the absorption ratio, surface plasmonic resonance peak intensity, and the second harmonic generation process. Finally, it also alters the so-called epsilon-near-zero (ENZ) effects, where the interplay between real and imaginary parts of permittivity results in the strong enhancement and local confinement of the normal field inside ENZ materials.^10,11^ Metals exhibit ENZ behavior in the visible and ultraviolet spectra and are building blocks for making ENZ metamaterials. However, lack of tunability and high losses in metals are critical factors limiting their applications.^12^

The growth mechanism and tunability of metallic nanoparticles are directly related to their fabrication methodology. Compared to other decoration methods with nanoparticles, such as chemical vapor deposition and liquid-state synthesis, physical vapor deposition (PVD) methods are simple to apply for nanoparticle decorations,^13^ and an in situ growth can easily be realized. Also, the PVD process usually does not involve using toxic or corrosive substances. Furthermore, the influence of reducing agents, precursors, and solvents, which are heavily involved in other methods, can be ignored. However, the growth mechanism of nanoparticles by PVD is dominated by adatom diffusion and aggregation along the sample surfaces. It is well known that PVD methods have less control of the morphology of nanoparticles than chemical methods.^13^ In this study, we have demonstrated that strain engineering could be an alternative route to control the morphology of metallic nanoparticles produced using PVD methods. AuNPs with different morphologies and optical properties can be deposited on the surface of the same perovskite thin film following a careful substrate selection. The AuNP growth mechanism could be controlled by the strain propagating from the substrate through the thin film to the AuNPs.

A strain is generated if a lattice mismatch exists between a thin film and a substrate. When the mismatch is less than approximately ±7%, a thin film grows following the lattice-matching epitaxy.^14^ Also, strain relaxation via dislocations is not energetically favorable at the interface. Depending on the material and substrate selection, some films require thicknesses of approximately 100–300 nm to allow the residual strain to be fully released.^15−17^ It is also the case that the relaxation of strain is not consistent with the increase in film thickness. The film’s lattice tends to match the substrate’s lattice at the film/substrate interface instead of relaxing the residual strain. Such a lattice-locking effect limits strain relaxation by lattice rearrangement. This effect is dramatic when the thickness of films is less than 50 nm and weakened for thick films.^18^ The differences in the thermal expansion coefficient can also introduce residual strain during the fabrication processes.^15,19,20^ Our recent research shows that strontium molybdate (SrMoO_3_) has a tunable strain-dependent permittivity crossover point.^16^ Residual strain influence on other properties has also been widely observed in other perovskite materials, e.g., the band gap energy of barium stannate, BaSnO_3_,^21^ and the magnetic properties of strontium ruthenate, SrRuO_3_.^22^

The strontium niobate (SNO) system consists of layers of Sr*_n_*Nb*_n_*O_3*n*+2_ with various oxygen concentration ratios. Its conductivity is highly sensitive to the molar ratio among elements.^23^ Different molar ratios distort the SNO unit cells from cubic to orthorhombic. A rising oxygen concentration generates planar defects with additional oxygen sites parallel to the *a*–*b* plane. These defects break the atomic periodicity in the *c*-direction, maintaining the *a*–*b* periodicity.^24^ The number of octahedral layers stacking between two planar defects equals the value of *n* in Sr*_n_*Nb*_n_*O_3*n*+2_. If there are no defects, *n* → ∞ and the material structure is SrNbO_3_ (SNO_3_) with *a* = 4.023 Å and holds atomic periodicities in *a*, *b*, and *c* directions.^25^ SNO_3_ is a plasmonic metallic oxide with an absorption band gap of around 1.9 eV.^26^ For *n* = 5, SrNbO_3.4_ (SNO_3.4_) shows a semiconducting behavior. When *n* = 4, SrNbO_3.5_ (SNO_3.5_) behaves as an insulator. Chen et al. reported that such a dependency is related to the change in the Nb atom valency.^27^ From their scanning transmission electron microscopy (STEM)-image-based density-of-states calculations, the conducting electrons in SNO_3_ are mainly contributed by Nb 4d orbitals. Splitting of the octahedral layers provides additional oxygen atom sites. Forming new oxygen–niobium bonds changes the Nb valance state from +4 to +5 and lowers the Fermi level from the conduction band to the valence band.

Here, we studied the formation of gold nanoparticles (AuNP) grown by pulsed laser deposition (PLD) onto perovskite SNO thin films deposited on both magnesium oxide (MgO) and strontium titanate (STO) substrates. The strain-free SNO_3_ (*a* = 4.023 Å) has a −3.0% lattice mismatch with the STO (*a* = 3.905 Å) and 4.5% with MgO (*a* = 4.212 Å), which leads to compressive and tensile in-plane residual strains, respectively. We found that the formation of AuNPs and their morphology highly depend on the lattice parameter of the underlying SNO film. It conveys the surface structure and controls nanoparticle formation. The morphologies of AuNPs were measured using SEM and atomic force microscopy (AFM). The residual strain in SNO thin films and AuNPs was evaluated by X-ray diffraction (XRD). Finally, the complex permittivity of AuNP-decorated SNO films was measured using variable angle spectroscopic ellipsometry (VASE).

## Methods

### Thin Film Fabrication

Samples were prepared using a multitarget PLD system (Neocera). Two substrates were selected to investigate the influence of the residual strain on sample properties. MgO substrates (Crystal 1MGO 106E, 5 × 5 mm^2^, (100) oriented, one side polished) and STO substrates (Crystal 1STO 105E, 5 × 5 mm^2^, (100) oriented, one side polished) were used. To ensure similar growth conditions in every deposition process, two substrates (STO and MgO) were mounted on a sample stage next to each other. The chamber was purged with nitrogen gas three times before the deposition process.

SNO_3_ films were fabricated using an in-house-manufactured ceramic SNO_3.5_ target. The target was prepared by mixing SrCO_3_ (≥99.9%, Sigma-Aldrich) and Nb_2_O_5_ (≥99.999%, LTS Research Laboratories, Inc.) in the appropriate stoichiometric proportions and then milled to ensure thorough mixing. The powder mixture was then calcined at 950 °C for 48 hours. The calcined media was then grounded in the same mill until a fine powder was obtained. The powder was pressed in a 2 cm diameter die at 190 MPa. The resulting powder compact was then sintered at 1100 °C for 24 hours. The XRD scan of the target confirmed that only the SNO_3.5_ single phase was obtained (see Figure S1). Gold nanoparticles were fabricated from a gold target (99.999% purity). A KrF (248 nm, COMPex) laser excimer operated with 10 Hz, 350 mJ was used. Substrates were heated to 800 °C, and the chamber pressure was maintained below 5 × 10^–6^ Torr. Between every twenty pulses (20 P) of SNO deposition, a 10 s dwelling time was introduced. Such a growth condition allows SNO atoms to form a fine crystal kinetically.^28^

Three groups of samples were fabricated to investigate the influence of substrates. The first group of samples was used to investigate the processes of the residual strain generation and relaxation due to the SNO/substrate lattice mismatch. This was realized by depositing SNO films of various thicknesses: 20, 40, and 80 nm. The second group of samples was used to reveal how the residual strain of SNO thin films affects the growth of AuNP. The exact amount of Au was deposited onto SNO layers with various thicknesses: 20, 40, 80, and 200 nm. The last group of samples explored the growth mechanism of AuNPs on the SNO thin film surface. Various amounts of gold, controlled by the number of laser pulses shot on the Au target (e.g., 250 P, 750 P, 1250 P, and 1750 P) were deposited on 20 nm SNO thin films grown on MgO and STO substrates.

### Thin Film Characterization

Nanoparticle shapes were characterized by SEM. SEM images of AuNPs on SNO surfaces were captured using an LEO Gemini 1525 field emission gun-SEM (FEG-SEM) instrument. Electron high-tension voltage was set at 5.00 keV, and an in-lens detector was selected. The lattice parameters of the samples were evaluated using XRD. An Empyrean multipurpose diffractometer instrument embedded with a Cu Kα X-ray source was employed. θ–2θ XRD scan was used to measure the out-of-plane lattice parameters of SNO thin films and AuNP. The 2θ–ω scanning mode was employed to plot reciprocal space mappings (RSMs) and characterize the in-plane lattice parameters of SNO samples. XRD results were further analyzed using X’pert HighScore Plus software and with relevant calculations to investigate the residual strain in the samples. The average roughness of SNO surfaces and the height of AuNPs were measured by AFM using an Asylum MFP-3D classic instrument. General-purpose silicon probes with an aluminum reflective coating (NuNano SCOUT 70 RAl) were employed for the tapping mode scans.

The permittivity was measured using a Woollam V-Vase ellipsometer at 300–1600 nm. The angles of incident light for measuring reflection were set at 65, 70, and 75°. The substrate contribution to the overall permittivity was excluded by fitting the collected data to a constructed substrate model. The model considered a substrate with a known permittivity value and an additional general oscillator (GenOsc) layer. The GenOsc layer contained one Drude and several Lorentz oscillators. These oscillators simulated the Drude resonance and interband transitions of electrons within SNO layers. Additional Lorentz oscillators were added to account for the interband transition in AuNPs.

## Results and Discussion

### Strain Relaxation in SNO Thin Films

**Figure 1:**
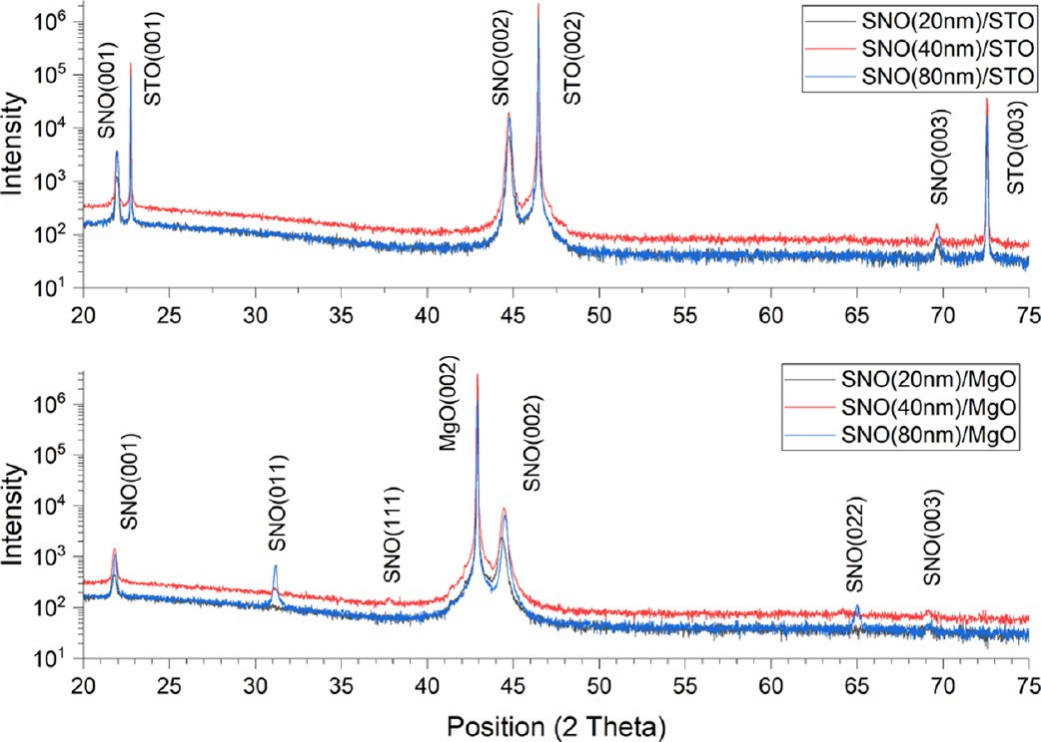
XRD patterns of SNO thin films with various thicknesses grown on STO and MgO substrates.

SNO thin films with various thicknesses were deposited on MgO and STO substrates. The thickness of the SNO films was measured by AFM and further confirmed by ellipsometry measurements. XRD was used to investigate the influence of substrates on the crystal structures of SNO layers. The peak positions of films and substrates on the θ–2θ XRD patterns (see Figure 1) confirmed the dominant epitaxial growth of SNO layers on both types of substrates. Also, they indicate that the grown SNO films are highly (00*l*) oriented. Shifts in the (002) peak position of SNO films on STO and MgO substrates are also observed. For example, there is a 0.05 and 0.16 2θ degree right shift of the (002) peak on STO and MgO substrates when the thickness increases from 20 to 80 nm, which reveals the change in the lattice parameters of thin films and residual strain relaxation from 0.6 to 0.5% on STO and 1.5 to 1.0% on MgO substrates. Additional SNO (111) and (011) peaks were detected on 40 and 80 nm SNO thin films grown (only) on the MgO substrate. The appearance of phases with such orientation is also associated with the thin film’s thickness-related residual strain relaxation process.

The c-lattice parameters of SNO samples were calculated using the Nelson–Riley extrapolations (shown in Figure S2a,b). Evaluated c-axis lattice parameters as a function of the film thickness are shown in Figure S2c. Both SNO samples on MgO and STO substrates have larger out-of-plane lattice parameters than the unstrained SNO_3_ unit cell. As film thickness increases, the c-lattice parameters decrease. And this trend can be regarded as a strain relaxation process by atom rearrangement. For thicker films, it is easier for atoms to rearrange themselves for a lower overall residual strain. One should also note the measurement uncertainty associated with XRD measurements of 20 nm films on both substrates. This is due to the broadened SNO peaks and weak SNO (003) peaks. Nevertheless, the thickness-related change in the lattice parameters of SNO films on STO substrates is well pronounced, while for the MgO substrate, it is within the range of the measurement error.

**Figure 2:**
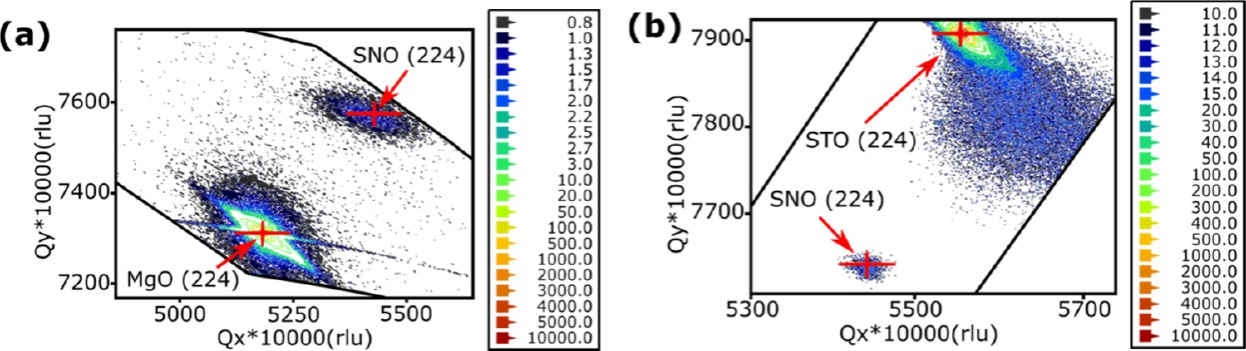
RSM plots of SNO thin films with 80 nm thickness grown on (a) MgO and (b) STO substrates. The mapping is plotted using the 2θ–omega XRD scanning mode. The (224) planes of the SNO samples and substrates are detected and potted on RSMs. The selected *q*-positions indicate that the SNO sample on the MgO substrate has a and b lattice parameters equal to 4.021 Å and the c-lattice parameter is equal to 4.068 Å (a). When grown on the STO substrate, the sample has *a* = *b* = 3.972 Å and *c* = 4.041 Å (b).

The reciprocal space mappings (RSMs, see Figure 2) detected the same tensile strain in the *c*-direction, and the RSM results also suggest that 80 nm SNO thin films grown on the STO substrate experience a compressive strain with a reduced *a* and *b* lattice parameter equal to 3.972 Å. The unit cell volume decreased by 2.1% from 65.11 Å^3^ in unstrained SNO_3_ to 63.74 Å^3^. The in-plane lattice mismatch between SNO and STO is reduced from −3.0% in unstrained SNO_3_ to −1.7% (see Table S1). However, the 80 nm SNO sample on the MgO substrate is fully relaxed in the *a, b* direction. There is negligible (0.04%) in-plane strain with *a* = *b* = 4.021 Å. The overall cell volume has increased by 1.0% to 65.78 Å^3^. Compared with the sample on the STO substrate, the MgO substrate does not influence the in-plane lattice parameters of the SNO film. The detected SNO (111) orientation is regarded as an in-plane strain relaxation mechanism due to the different signs and magnitude of the lattice mismatch.

**Figure 3:**
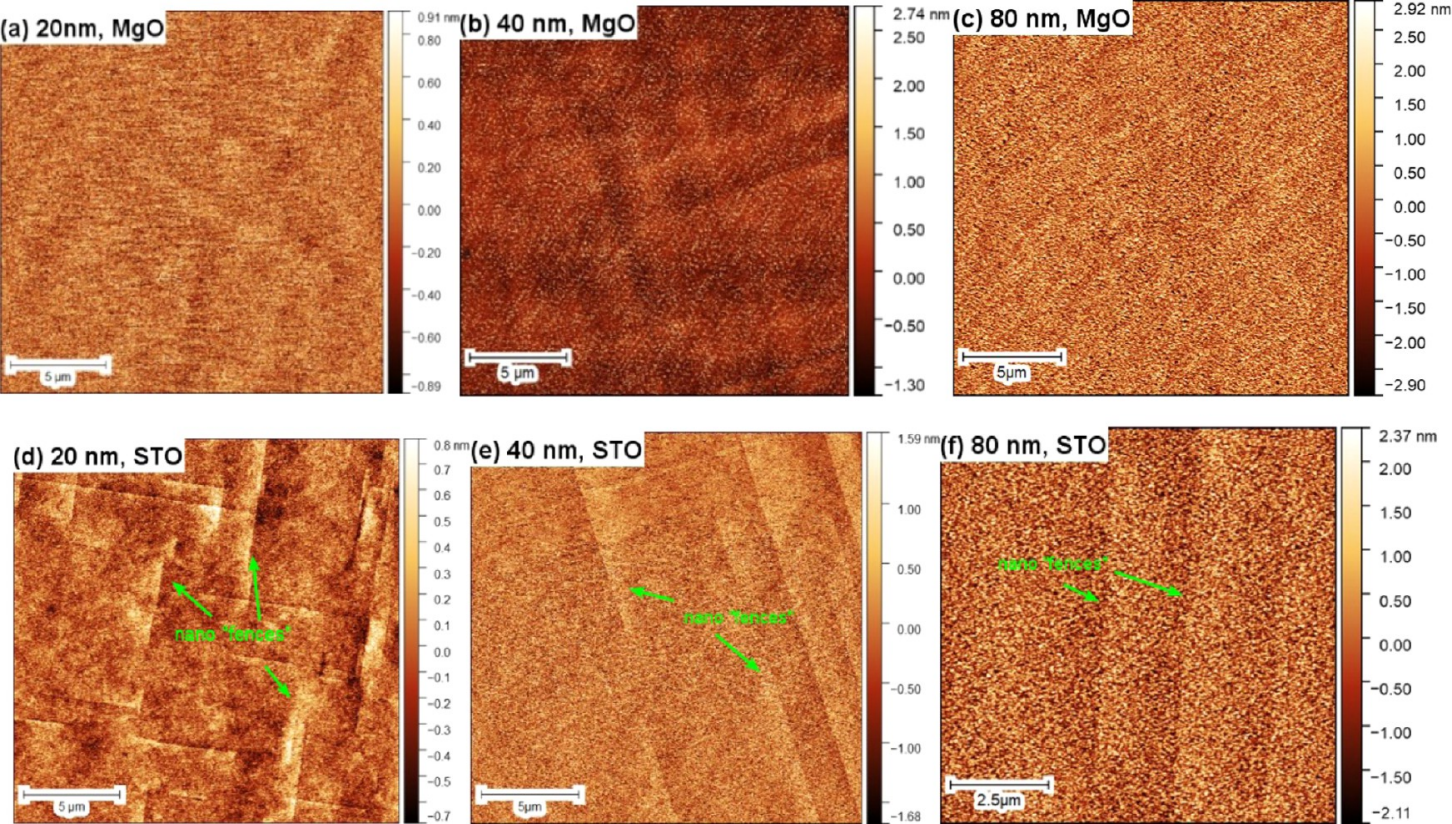
Morphology of 20 μm × 20 μm SNO thin film surfaces grown on MgO substrates with thicknesses of (a) 20 nm, (b) 40 nm, and (c) 80 nm; and on STO substrates with thicknesses of (d) 20 nm, (e) 40 nm, and (f) 80 nm. The green arrows in panels (d–f) show the nano “fences” observed on SNO surfaces.

The surface roughness of SNO thin films was characterized by AFM (see Figure 3). The root-mean-square roughness values of SNO/MgO surfaces are 0.3, 0.6, and 1.0 nm for layers with thicknesses of 20, 40, and 80 nm, respectively. SNO thin films grown on STO substrates result in slightly lower roughness values of 0.2, 0.5, and 0.7 nm, respectively. Regardless of the substrate selection, the surface roughness of SNO thin films increases with increasing film thickness. This trend further proves that atoms are locked near the SNO/substrate interfaces. These localized atoms are less responsive to the strain generated at the interface and release the strain ineffectively by rearranging themselves. When the thickness of the film increases, the residual strain can be freely released. This is accompanied by increased surface roughness. Also, nano “fences” are observed on SNO/STO surfaces. The height of these fences is between 0.5 and 1 nm. The origin of these “fences” is the boundary where two nucleation domains meet.

### AuNP Growth Mechanism on SNO Thin Films

**Figure 4:**
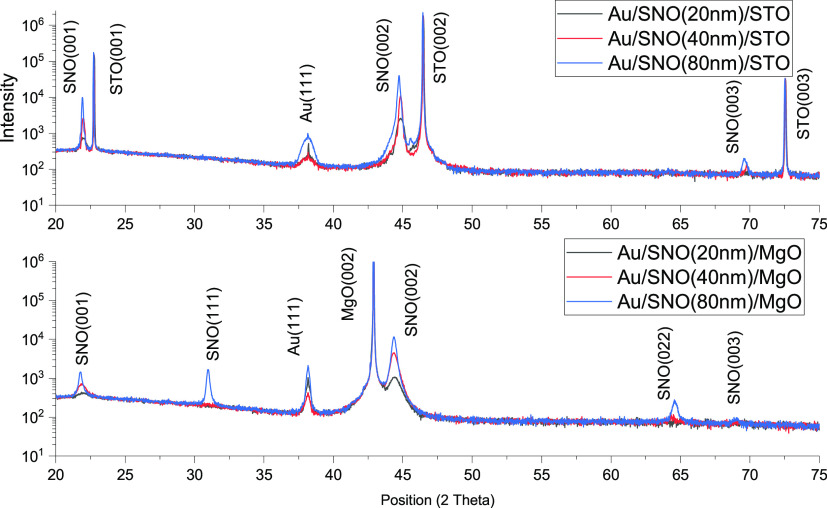
θ–2θ scan of 1250 P AuNP-decorated SNO thin films with various thicknesses.

As discussed in the previous section, the amount of gold deposited on the surfaces of SNO thin films was controlled by the number of laser pulses shot on the Au target. Figure 4 shows the XRD patterns of 1250 P Au grown on 20, 40, and 80 SNO thin films. The type of substrate and SNO thickness have a limited impact on the Au lattice parameter as Au XRD peak positions do not shift and have negligible strain compared to the reference. The lattice parameters evaluated for all structures are shown in Figure S3. Compared with samples without Au decoration, the decorated SNO unit cells show a similar value in their c-lattice parameters, which is in the range of 4.070–4.085 Å on MgO and 4.035–4.045 Å on STO substrates. It is worth noting the different trend in the thickness-related SNO film lattice parameters before and after AuNP decorations. However, one could argue that it is still within the range of the lattice parameter evaluation error.

**Figure 5:**
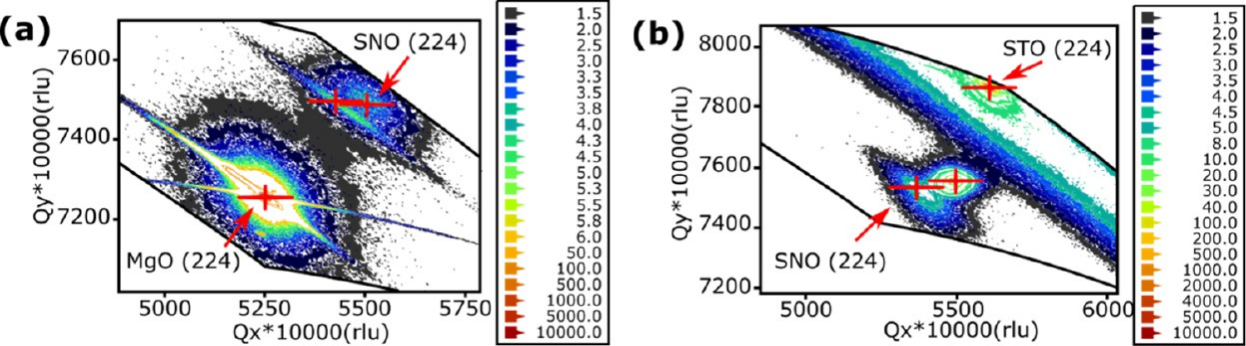
RSM plots of SNO thin films with 80 nm thickness grown on (a) MgO and (b) STO substrates. Both plots indicate that SNO shows two (224) peaks after the AuNP decoration.

Compared to the RSM plots of undecorated SNO films (see Figure 2), both the AuNP-decorated thin film’s RSM plots (see Figure 5) exhibit an unexpected splitting of SNO (224) peaks. The spreading of the (224) peak can be attributed to the mosaic spread and strain gradient within the film. This peak splitting shows an additional misorientation that appears after the AuNP decoration. It is likely that Au/SNO interface acts as a second strain source for the SNO thin film. It is worth noting that the AuNP is deposited at a high temperature. Therefore, the strain could be due to different thermal expansion ratios or partial SNO film recrystallization. The sandwiched (SNO) layer is affected by the stresses generated at its bottom (substrate–film) and top (film–AuNP) interfaces that change its residual strain relaxation and distribution. A similar unit cell alternation in a sandwiched perovskite-type thin film was previously observed and reported by Petrov et al.^29^

**Figure 6:**
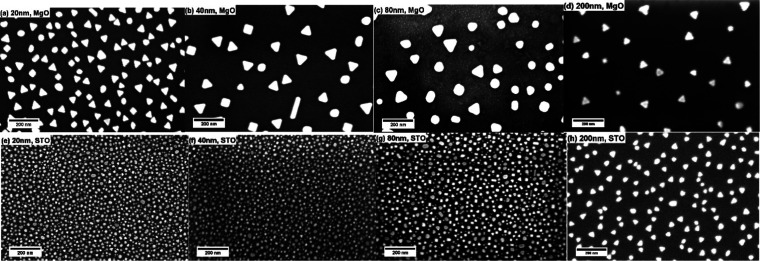
SEM images of 1250 P of Au plume deposited on (a) 20 nm, (b) 40 nm, (c) 80 nm, and (d) 200 nm SNO layers on MgO substrates; and (e) 20 nm, (f) 40 nm, (g) 80 nm, and (h) 200 nm SNO layers on STO substrates.

The morphologies of the deposited AuNPs were examined using SEM and shown in Figure 6. On the SNO/MgO surface, AuNPs present distinct corners and edges, which are evidence of the preferred direction of growth during its growth process. As a comparison, AuNPs grown on SNO/STO surfaces exhibit a dramatically different morphology. The size of these AuNPs is smaller, while their number density and surface coverage ratio are significantly increased. The shape of the AuNPs on SNO/STO surfaces is rounded. The results of the size analysis of AuNPs are shown in Figure S4. For 20, 40, 80, and 200 nm SNO thin films, the size distribution for AuNPs on SNO/MgO is unimodal with average sizes of 1900, 3500, 2900, and 2500 nm^2^, respectively. The size of AuNP decreases with an increased thickness except for the 20 nm sample. In contrast, there is a bimodal size distribution for the AuNPs on SNO/STO for 20–80 nm SNO thin films. The average sizes of the larger AuNPs are 250, 280, 440, and 1040 nm^2^ on 20, 40, 80, and 200 nm SNO thin films, respectively.

**Figure 7:**
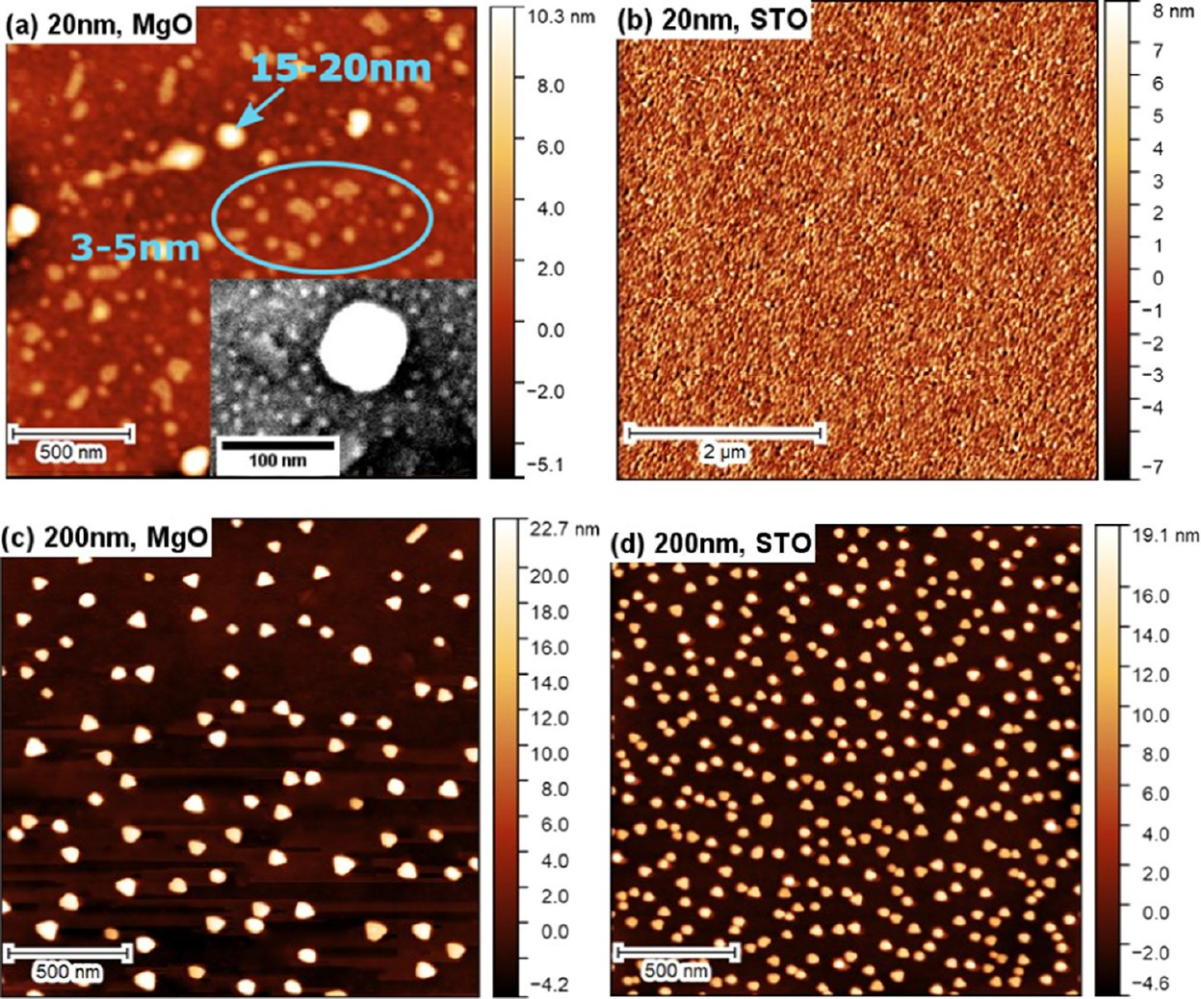
AFM images of the surface of AuNP-decorated 20 nm SNO thin films on (a) MgO and (b) STO substrates. (c, d) Decorated 200 nm SNO thin films using MgO and STO substrates, respectively. The inserted SEM image in panel (a) is captured from the surface of the AuNP-decorated 80 nm SNO thin film grown on the MgO substrate. The average height of AuNPs in panel (c) is 26.7 nm and that in panel (d) is 18.5 nm using MgO and STO substrates, respectively.

The shape of AuNPs on SNO surfaces was further studied by AFM. In Figure 7a,b, the average height of AuNPs can be measured. Surprisingly, two types of AuNPs can be observed on the SNO/MgO surfaces. These features were not detected during the SEM and size analysis. The larger AuNPs have an average height between 15 and 20 nm, while the smaller ones are between 3 and 5 nm. Similar small nanoparticles are only observed at very high magnified (300kx) SEM images of 80 nm SNO/MgO samples (shown as an inserted SEM image in Figure 7a). AuNPs on SNO/STO substrates have an average height of about 5 nm across the sample surface, which is similar to the height of the small AuNPs on SNO/MgO surfaces. The residual strain in the SNO layer can explain this phenomenon.

The influence of stress at the SNO/substrate interface on the AuNP/SNO interface can be minimized by increasing the thickness of the sandwiched SNO layer. As the thickness of the SNO layer increases, the substrate’s lattice-locking effect is mitigated. The SEM and AFM results confirm that the increase in thickness leads to a larger AuNP area on SNO/STO surfaces. To further prove this hypothesis, AuNPs were deposited on 200 nm SNO thin films grown on both substrates. It is found that the growth mode of AuNPs is becoming similar on both surfaces (see Figure 6d,h for SEM images, Figure S4 for the size analysis, and Figure 7c,d for AFM results). AuNPs grown on both SNO surfaces have unimodal size distribution with a preferred direction of growth. No small AuNPs are observed on the AFM images. The size analysis also shows that the size of NPs on MgO decreased, while an opposite trend is observed for the samples on the STO substrate. Their average sizes approach parity as the thickness increases. However, AuNPs grown on SNO/STO surfaces still have a smaller average size and higher NP density. This indicates that the strain is not fully relaxed even at 200 nm thickness.

**Figure 8:**
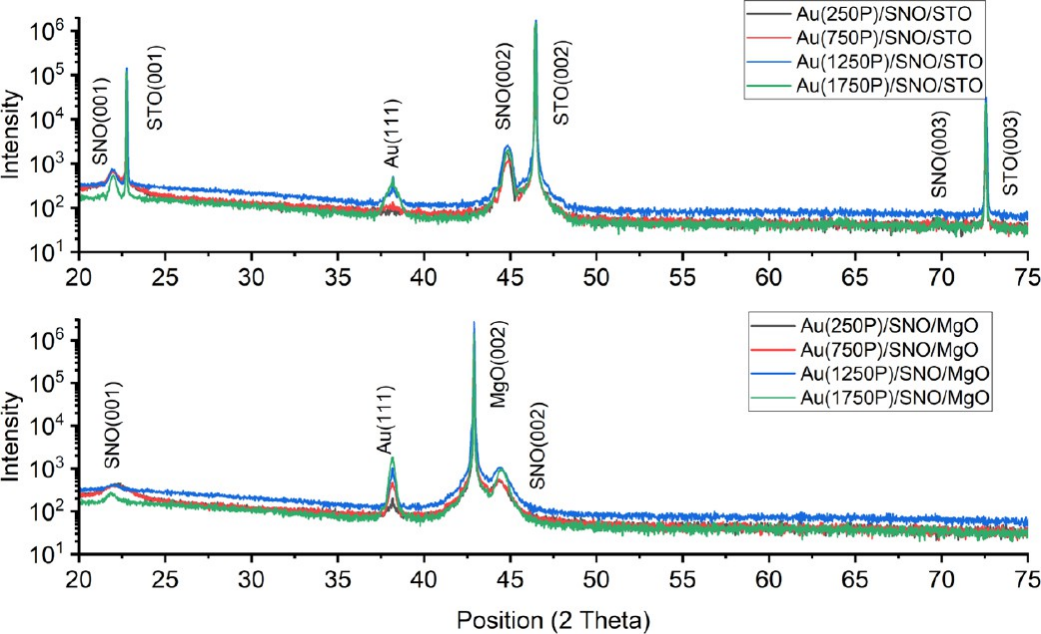
XRD θ–2θ scans of different amounts of AuNP-decorated 20 nm SNO thin films.

The growth of AuNPs on SNO thin film surfaces was further investigated by depositing various amounts of Au on 20-nm-thick SNO films. The measured XRD patterns are shown in Figure 8. The patterns are similar to those presented in Figure 4. As mentioned in the previous section, there is a negligible out-of-plane residual strain in AuNPs calculated from XRD peak positions. The normalized area under Au (111) peaks can effectively reflect the size of AuNPs on the SNO surfaces. Extremely poor Au peaks were detected after 250 P and 750 P of Au deposition on SNO/STO samples (see Figure 8). The area under the Au (111) peak increases by increasing the amount of deposited gold. Also, it has to be noted that the XRD patterns of the AuNP-decorated SNO/STO samples contain both sharp and broad peaks, revealing a broad NP size distribution. In contrast, the Au (111) peaks in the XRD patterns of the AuNP-decorated SNO/MgO samples have a smaller FWHM value and a larger peak area, indicating a different growth mechanism to those on the STO substrates.

**Figure 9:**
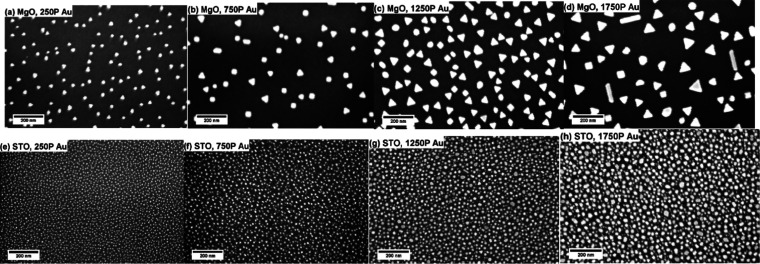
SEM images of AuNPs deposited on the SNO (20 nm)/MgO surface for varying deposition laser pulses of (a) 250 P, (b) 750 P, (c) 1250 P, and (d) 1750 P; and on the SNO (20 nm)/STO surface for (e) 250 P, (f) 750Ps, (g) 1250 P, and (h) 1750 P.

SEM images of AuNPs on SNO surfaces are shown in Figure 9. Their shapes are similar to those shown in Figure 6. SEM image-based size distribution analysis of AuNPs is presented in Figure S5. AuNPs grown on SNO/MgO surfaces have average sizes of 640, 1400, 1900, and 2800 nm^2^ for 250 P, 750 P, 1250 P, and 1750 P of Au deposition, respectively. The increased Au deposition time results in a larger NP size and a broader size distribution. For each increment of 500 pulses, the mean area grows ∼700 nm^2^, suggesting that the growth mechanism is consistent throughout the experiment. On SNO/STO surfaces, the averaged sizes of the larger AuNPs are too small to be measured for 250 P, and 135, 255, and 470 nm^2^ for 750 P, 1250 P, and 1750 P of Au deposition, respectively. These values are smaller than those measured on MgO substrates, which agrees with the XRD peak area analysis. Owning to the resolution of SEM images, the discussion of the size of smaller AuNPs is not meaningful. These SEM images clearly show the different favored growth directions of AuNPs on the two surfaces. Compared to SNO/MgO surfaces, AuNP growth on the SNO/STO surface is much more preferred, reflected by a dramatically higher surface coverage. This result also solidifies the statement that residual strain in SNO thin films could affect the diffusion of Au adatoms along the SNO surfaces. During the nucleation stage, Au adatoms on the SNO/STO surface cannot reach the nearest nuclei before the next pulse comes owing to a slow diffusion speed. These slowly diffused adatoms aggregate with incoming Au, reach supersaturation, and form agglomerates. This is reflected in its round-shaped, high number density, and high surface coverage ratio by AuNPs. Large NPs on both surfaces are grown via fast-diffused Au adatoms. These AuNPs exhibit distinct edges and corners because they are grown via the diffusion of adatoms along the surface instead of from the Au plume. This triangular shape suggests a single crystal structure of AuNPs.

### Influence of the Substrate on Optical Properties

**Figure 10:**
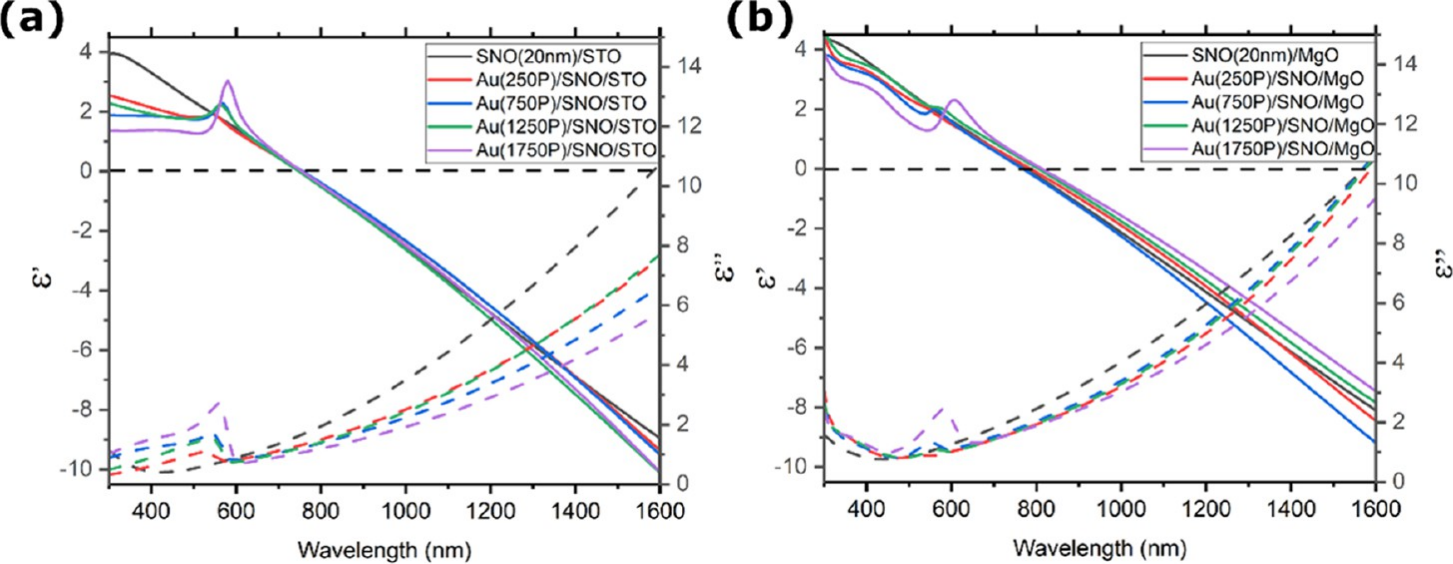
Complex permittivity of AuNP-decorated 20 nm SNO thin films. Solid and dashed lines represent the real and imaginary permittivity of samples, respectively.

Ellipsometry is used to analyze the optical properties of these AuNPs with different morphologies. Their complex permittivity within the 300–1600 nm wavelength region is shown in Figure 10. SNO films grown on STO substrates are more metallic than those on MgO substrates, proved as a larger absolute ε′ value at the near-infrared region. This difference suggests lower lattice distortions, e.g., oxygen vacancies and NbO_6_ octahedral tilts, and agrees with the XRD and AFM results shown and discussed above. The AuNPs show a broad peak in the <600 nm region, which is expected from the Au 5d to 6s interband transition. This is most evident in the SNO/STO samples with the smallest AuNPs. In the 530–600 nm range, the effect of the AuNP plasmonic resonance is noticeable. The SNO/STO samples producing smaller AuNPs have resonances nearer 530 nm, while the SNO/MgO samples with larger AuNPs create resonances nearer 600 nm. The breadth and variation in this absorption across the samples could be attributed to the change in the AuNP size distribution, shapes, or NP densities. There is also a consistent red shift of the resonance position as the deposition time of AuNPs increases, reflecting the increases in AuNP size and density. Meanwhile, the AuNP decoration has a limited impact on the real part of SNO layers’ permittivity (ε′). The epsilon-near-zero crossover wavelength (745 nm) for the SNO/STO substrate is not affected by AuNP decorations. In comparison, AuNPs deposited on SNO/MgO surfaces shift the crossover wavelength by 50 nm from 765 to 815 nm. In the latter case, the ε′ value also becomes more dielectric as more Au is deposited. Remarkably, AuNPs on both surfaces have a more profound impact on the imaginary part of permittivity (ε″), especially in the NIR region. AuNP-decorated SNO/STO samples have a notably lower loss than those of SNO/MgO samples. This may be because AuNPs prevent optical signal penetration into the underlying SNO film. This is also consistent with the changing ε′ value in the near-infrared region.

## Conclusions

AuNPs were deposited on SNO thin films grown on two substrates with distinct lattice parameters (MgO and STO), which resulted in the build-up of residual strain with opposite signs. The type and value of the residual strain do not play a significant role in the growth of SNO thin films. However, it dramatically affects the morphologies of AuNPs grown on SNO films. AuNPs grown on SNO/STO surfaces have small sizes, rounder shapes, and high number density. In contrast, AuNPs on SNO/MgO surfaces are larger and exhibit distinct edges and corners. SNO thin film samples decorated with AuNPs with different morphologies also show changes in optical properties as expected. AuNP-decorated SNO/STO samples display a more metallic behavior and a broad interband transition. They also have a lower optical loss in the NIR region. In contrast, the MgO samples exhibit a distinct interband transition.

The change in AuNP morphologies and growth mechanisms originates from the strain produced at SNO/substrate interfaces, which is conducted by SNO thin films, and finally impacts the diffusion length of Au adatoms along the surface. Since SNO grown on MgO has a closer lattice parameter to Au, it is energetically favorable for Au to diffuse along the surface. Therefore, AuNPs grown are larger and show a triangular shape based on the favored direction of growth. On SNO/STO surfaces, slow-diffused Au adatoms aggregate with the adatoms arriving at the surface. These clusters reach supersaturation and form small and rounded agglomerates. The influence of the strain depth introduced by the substrate can be mitigated by increasing the thickness of the sandwiched SNO layer. A similar type of AuNPs with triangular shapes was fabricated on the surface of 200 nm SNO thin films, regardless of the substrate selection.

Thus, we propose an additional method to alter the growth dynamics of AuNPs decorating thin films. The shape and size of the AuNP can be tailored by controlling the residual strain of thin films. This method could be used to assist the development of plasmonic samples with desired optical properties for biosensing, photocatalysis, and solar cell applications.

## Supporting Information Available

θ–2θ scan of SNO target; Nelson–Riley extrapolation plots of the c-lattice parameters of SNO/AuNP thin films; size analysis of AuNPs grown on SNO thin films with various thicknesses; and SEM image-based AuNP surface area analysis (PDF)
