# Supplementary material for: Crystalline AuNP-Decorated Strontium Niobate Thin Films: Strain-Controlled AuNP Morphologies and Optical Properties for Plasmonic Applications
Source: ACS Appl Nano Mater. 2023 Jun 20;6(13):11115–23. doi: 10.1021/acsanm.3c00934 (PMC10352961; doi:10.1021/acsanm.3c00934)
Supplement: Supplementary file 1 — an3c00934_si_001.pdf [file an3c00934_si_001.pdf]

Crystalline AuNP Decorated Strontium Niobate Thin Films: Strain-controlled AuNP Morphologies and Optical Properties for Plasmonic Applications

Qiaomu Yao[1], Andrey V. Berenov[1], Ryan Bower[1], Bin Zou[1], Xiaofei Xiao[2], Neil M. Alford[1], Rupert F. M. Oulton[2], and Peter K. Petrov*[1]

1. Department of Materials, Imperial College London, London SW7 2AZ, United Kingdom
2. Department of Physics, Imperial College London, London SW7 2AZ, United Kingdom

Supplementary Information

XRD analysis

The strontium niobate target ($SrNbO_{3.5}$) was prepared by mixing $SrCO_3$ and Nb_2O_5 powders. XRD measurement was used to confirm its final composition.

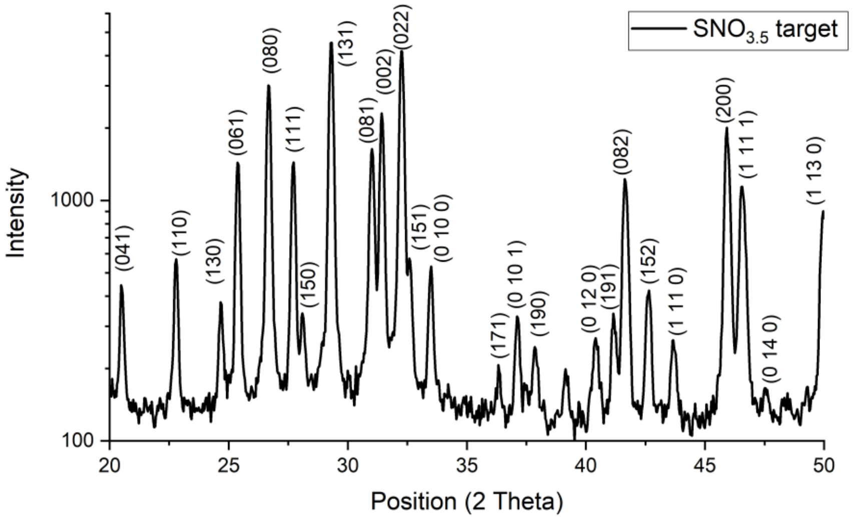

Figure S1: The θ-2θ scan of SNO target. The pattern confirms that a $SNO_{3.5}$ single phase was fabricated.

The lattice parameters of SNO thin films were evaluated from their XRD peak positions using the Nelson-Riley extrapolation function. The evaluated lattice parameter was then compared with the reference value to calculate the residual strain.

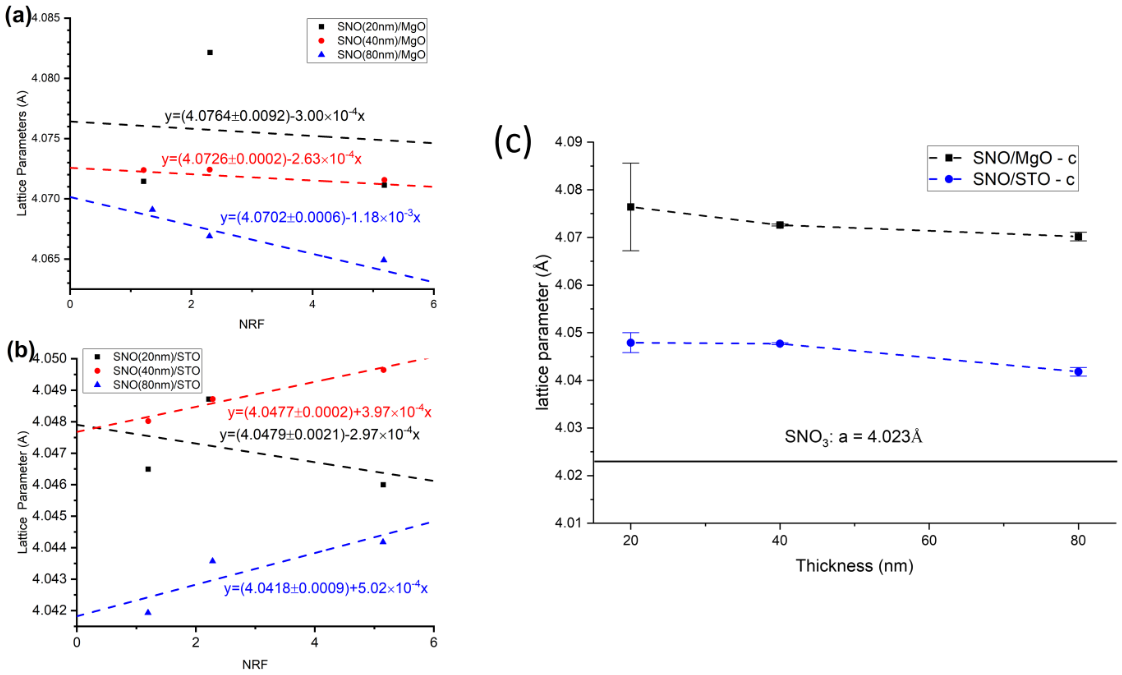

Figure S2: The Nelson-Riley Extrapolation plots of the c-lattice parameters of SNO thin films deposited on (a) MgO and (b) STO substrates. (c) SNO c lattice parameters as a function of the film thickness. The dashed lines represent the trend of c parameters change with film thicknesses. The solid black line represents a referential cubic SNO_3 unit cell, with a = 4.023 Å.

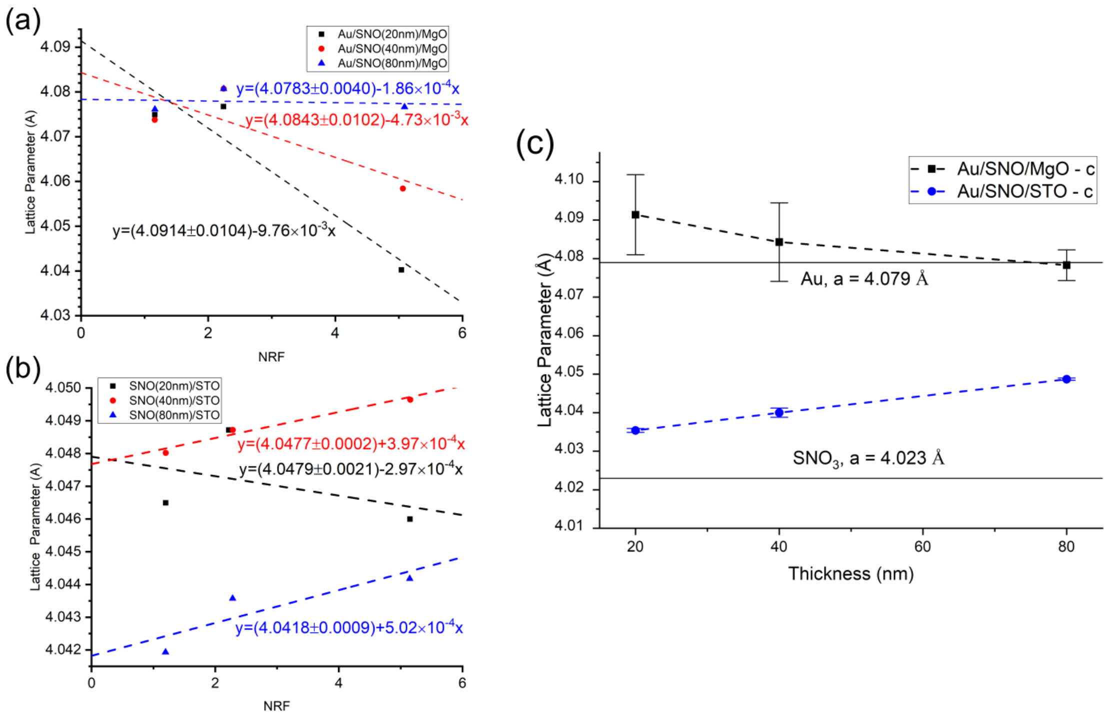

Figure S3: The Nelson-Riley Extrapolation plots of the c-lattice parameters of AuNP decorated SNO thin films deposited on (a) MgO and (b) STO substrates. (c)The c lattice parameters of SNO thin films are calculated from θ-2θ XRD patterns. The solid lines are the referential lattice parameters of Au and SNO_3.

To evaluate the in-plane lattice parameters, reciprocal space mapping was used to scan the SNO (244) peak. The residual strains were calculated by comparing their lattice parameters to the reference value.

Table S1: the lattice mismatch at SNO (80 nm)/substrate interfaces and the residual strain in SNO unit cell analysed from the XRD and RSM results.

Substrate	Lattice mismatch between SNO and substrate	Lattice mismatch from RSM results after relaxation	in-plane residual strain in SNO unit cell	out-of-plane residual strain in SNO unit cell	Change in SNO unit cell volume
STO	-3.0%	-1.7%	-1.2%	0.4%	-2.1%
MgO	4.5%	4.5%	Negligible	1.1%	1.0%

Gold nanoparticles size analysis

The size of gold nanoparticles was evaluated from their SEM images. ImageJ was used to distinguish the nanoparticles from the background based on brightness and contrast. The size of gold nanoparticles can be obtained using the software embedded 'analyse particles' function by counting the number of pixels. At least 5 SEM images at different locations were used for each sample.

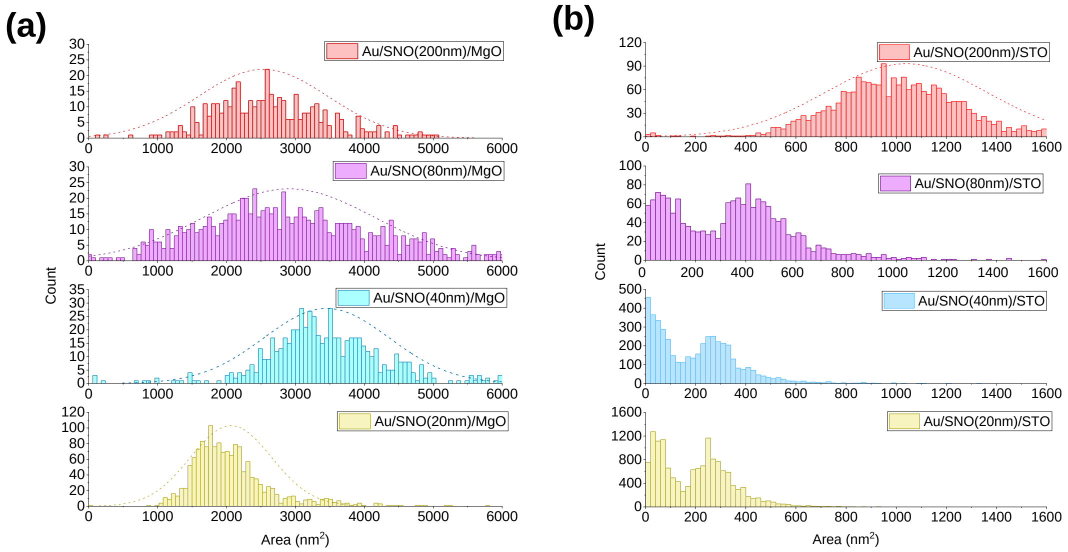

Figure S4: The size analysis of AuNPs grown on SNO thin films with various thicknesses. (a) The size distributions of AuNPs grown on SNO/MgO surface, fitted with a normal distribution curve (dashed line). (b) The size distributions of AuNPs grown on the SNO/STO surface is bimodal, representing the amorphous and crystalline AuNPs (when crystallising, AuNPs tend to have a bigger area).

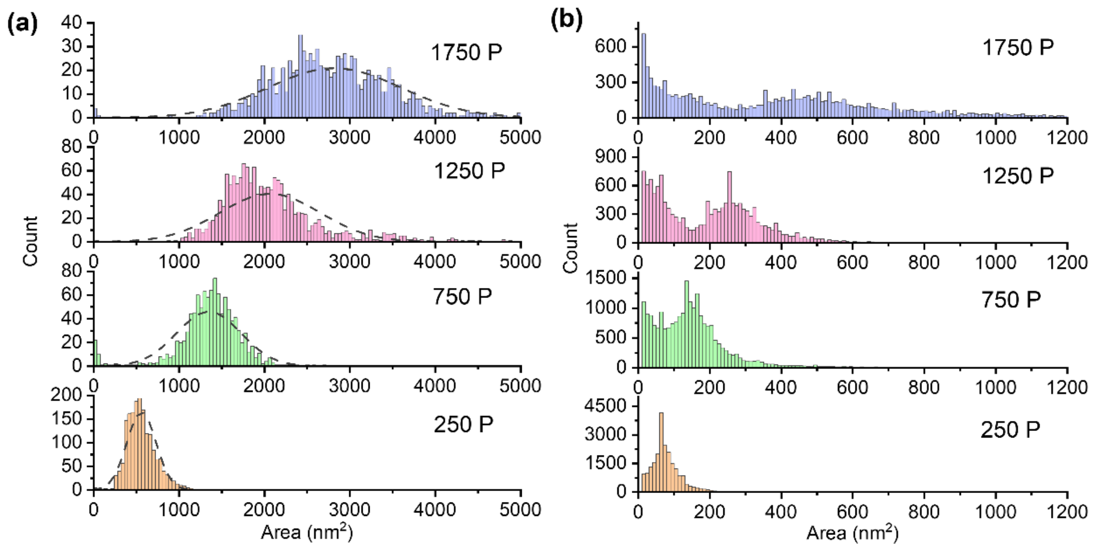

Figure S5: SEM image-based AuNPs surface area analysis. (a) The area distribution of AuNPs deposited on SNO/MgO surface with various depositing times. The dashed line is the fitted normal distribution. (b) The area distribution of AuNPs deposited on the SNO/STO surface (the deposition time is the same as the SNO/MgO samples).
